# Practical Status and Social Background of Current Mobile Stroke Units Worldwide: A Survey and Investigation

**DOI:** 10.5811/westjem.21267

**Published:** 2025-05-19

**Authors:** Masahiko Hiroki, Mototsugu Kohno, Yutaka Kohno, Masaki Misawa

**Affiliations:** * Tsukuba Medical Center Hospital, Department of Neurology, Tsukuba, Ibaraki, Japan; † Tsukuba Medical Center Hospital, Department of Emergency and Critical Care Medicine, Tsukuba, Ibaraki, Japan; ‡ Ibaraki Mobile Healthcare Corporation, Tsukuba, Ibaraki, Japan; § Ibaraki Prefectural University of Health Sciences, Department of Neurology, Center for Medical Sciences, Ami, Ibaraki, Japan; || Faculty of Health Sciences, Department of Radiological Sciences, Komazawa University, Setagaya, Tokyo, Japan

## Abstract

**Background:**

We aimed to clarify the current challenges involved in introducing and operating mobile stroke units (MSU) in new regions, considering the social background of regions with MSUs.

**Methods:**

We conducted a questionnaire survey on the operational and financial status of all active MSU programs worldwide as of March 2023, and investigated the demographic, economic, and healthcare backgrounds of areas with and without active MSUs. We compared the data for the two groups at the country, state, or city level. We then correlated data gathered from the survey and the investigation.

**Results:**

Of the 33 MSU programs contacted, 19 (59%) responded. The responding programs treated a range of 52–1,663 (median 781) patients at an MSU per year. The most commonly reported hours of operation were eight hours every weekday (5, 26%). The majority had four staff on board (11, 58%). No physicians were on board in six MSUs (32%). The catchment area radius ranged from 5–250 (median 22) kilometers. The start-up costs and subsequent annual operation costs of an MSU ranged from $0.7–1.8 million (median 1.0) and $0.7–1.7 (median 1.0) million US dollars, respectively. Reimbursement was obtained by eight (47%), with full reimbursement by two (12%). A negative gross financial balance was reported in eight MSUs (53%, of 15), and a financial challenge was reported in 17 (94%, of 18). Compared to the non-MSU group at the country level, active MSU groups had a significantly higher population, nominal gross domestic product, healthcare access and quality index, and physician density. They also had significantly lower age-standardized stroke incidence rates and age-standardized stroke disability-adjusted life year rate. The MSU operation time was significantly positively correlated with age-standardized stroke incidence rate and negatively with physician density.

**Conclusion:**

Despite facing serious financial problems, mobile stroke units currently operate around the world. However, the social context of MSUs appears relatively advanced. For future implementation of MSUs, cost-saving strategies and reimbursements should be addressed, and national or regional social backgrounds should be considered.

## INTRODUCTION

A mobile stroke unit (MSU) is a specialized ambulance equipped with a computed tomography (CT) scanner and telemedicine capabilities. The MSUs allow for the diagnosis and treatment of acute stroke patients at emergency scenes and enable prehospital triage to ensure that a patient is sent to the most appropriate hospital. The clinical usefulness of MSUs for tissue plasminogen activator treatment in ischemic stroke patients has been evidenced in terms of functional outcomes. 1,2 Similarly, studies have shown that MSUs are more cost effective compared to standard ambulances. 3,4 The MSUs can also save time from dispatch to mechanical thrombectomy in ischemic stroke patients, decreasing their odds of suffering disabilities. 5 In patients with intracerebral hemorrhage, MSUs facilitate ultra-early management or prehospital triage. 6

Since the first MSU was introduced in Homburg, Germany, in 2008, 7 the use of MSUs has gradually spread around the world. According to the updated world map of MSUs created by Fassbender et al, 8 there were 33 active MSU programs worldwide as of 2022 with an additional eight MSU programs in the planning or implementation stage. The distribution of MSUs appears to be concentrated in North America and Western Europe. Because stroke is the second leading cause of both disability and death globally, with the highest burden of the disease in the countries or regions of Africa and Asia, 9 the implementation of MSUs in such regions is expected. 8

However, despite the cost effectiveness of MSUs, the high cost of their installation and operation and the uncertainty of reimbursements pose a financial barrier. 10, 11 These issues create a significant obstacle to implementing MSUs, especially in remote and rural areas or in developing countries. 12, 13 For project teams planning to install and continuously operate MSUs in new regions, it is essential to develop a working organization and socio-technical infrastructure. 14 Additionally, it is crucial to update understanding of the current status of active MSUs worldwide and clarify their social background, since this has never been studied.

In this study, we conducted a global survey to update data regarding the current practical status of MSUs and a novel investigation to clarify the social backgrounds of regions with and without MSUs. The survey was conducted independently of the support of the Prehospital Stroke Treatment Organization (PRESTO [https://www.prestomsu.org/]), an international consortium of MSUs, as we sought to obtain information from non-PRESTO MSU programs, presenting ourselves as questioners exploring the implementation of and business plans for MSUs.^11^,^15^

## METHODS

### Study Design

#### Survey of Active Mobile Stroke Units

We emailed the survey to the 33 active MSU programs worldwide as of 2022. These program sites were identified through Fassbender et al’s 2023 review paper. 8 Contact information for the representative or director of each MSU program was obtained through the programs’ official websites.

In the e-mail sent to each MSU program, we provided a status update on our own planned project to implement MSUs in Japan; we requested current information and described our study goals. We then requested updated operational and financial information on the MSU program that was current as of March 2023. The survey comprised nine structured questions referring to a single MSU. Respondents could select answers from multiple choices or write their own responses. The requested information was as follows: 1) model name of onboard CT scanner (multiple choice); 2) time and hours of operation (free-text description); 3) average number of personnel on board (multiple choice); 4) catchment area (free-text description); 5) start-up cost and annual operating costs (free-text description); 6) financial source (multiple choice), 7) status of reimbursement (multiple choice), 8) gross financial balance (multiple choice), and 9) current challenges they face (multiple choice). Finally, respondents were given the option to provide comments in a free-text section. If MSU operations were suspended as of March 2023, we requested information on the most recent date of MSU operations.

**Population Health Research Capsule**

What do we already know about this issue?

*Mobile stroke units (MSU) facilitate the diagnosis and treatment of acute stroke patients in the emergency setting, but start-up and operational costs are high.*

What was the research question?

*What is the updated status of MSU implementation? What is the social background of regions with MSUs vs those without?*

What was the major finding of the study?

*There are 33 MSU programs, each treating 52–1,663 (median 781) patients per year. Start-up and annual costs were $0.7–1.8 million (median 1.0) and $0.7 –1.7 (median 1.0) million US dollars, respectively. Countries with MSUs had higher GDP (P< 0.0001; 99.99% CI, 2,328–7,998 billion USD) than those without MSUs.*

How does this improve population health?

*While MSUs improve health outcomes, cost-saving strategies and reimbursements must be addressed, along with social background, prior to establishing a MSU.*

The e-mails were delivered beginning March 7, 2023. Deadline for response was April 30, 2023. All responses went to one of the authors (MH). In some cases, a single e-mail reminder was sent. For the responding programs, a post-survey was additionally conducted to ascertain the number of patients treated per a single MSU per year, as referenced in their published papers or official websites.

#### Investigation of MSU Vs Non-MSU Regions

We collected the most recent data on all regions with an active MSU (33 units in 11 countries, 24 states, and 33 cities) to investigate demographic, economic, and healthcare backgrounds at the country, state, or city levels. Similarly, for non-MSU regions, data were collected from the countries, from other states in the same MSU country, and from other cities in the same MSU state. We gathered the data from comprehensive public sources via the internet. The data consisted of the following from the studied countries, 16 states, 17–19 and cities 18–20: 1) population; 2) population density; 3) nominal gross domestic product (GDP) of the studied countries 23 and states 18,24; 4) the age-standardized incidence rate of all strokes in the studied countries; 9 5) the age-standardized disability-adjusted life years (DALYs) of all strokes in the studied countries 25; 6) the healthcare access and quality (HAQ) index in the studied countries 26; and 7) physician density in the studied countries 27 and states. 18 In these citations, only key references were provided due to the limitation of the number of references.

To be included in this study, the countries had to have a population of at least one million. We made this decision due to the uncertainty surrounding the effectiveness of MSUs in countries with very small populations. “States” refer to administrative divisions, such as states, regions, provinces, or counties. The designation “cities” included the smallest administrative districts, in cases where the city data was unavailable. For non-MSU regions, city population, size, and density were collected from the top 20 most populous cities in the same state that contained an MSU site. This ensured validity in comparison of population size or density between the small number of MSU cities and the large number of non-MSU cities, the latter of which are often sparsely populated. If an MSU city was identical to the administrative district, we collected data for the non-MSU cities from the adjacent states.

### Statistical Analysis

We performed descriptive analysis on the survey responses to determine the current operational and economic status of the MSUs, and the number of patients treated in a MSU per year. Inferential analysis was also conducted to examine the relationship between the status variables, which included the model of onboard CT scanners, hours of operation, number of onboard personnel and physicians, the catchment radius, the start-up cost, the annual operating cost, the status of reimbursement, and the gross financial balance. We used the Spearman rank-order correlation test or Kruskal-Wallis rank-sum test for these analyses. To investigate the differences between active MSU and non-MSU regions, we compared the two types of locations across all demographic, economic, and healthcare background categories. At the country level, we used the Mann-Whitney U test. At the state and city levels, we performed the Wilcoxon signed-rank test with mean ranks and mean ranks of mean ranks, respectively. Additionally, we investigated a threshold of each category between active MSU and non-MSU regions at the country level. This was estimated as having a maximum F1 score, by using the receiver operating characteristic analysis and binomial logistic regression analysis. The threshold was finally indicated when a predictive probability of overall model quality was ≥0.60 and a predictive accuracy rate was ≥0.80. Finally, for the active MSU sites, all operational and economic status variables were correlated with all background variables of active MSU regions, using the Spearman rank-order correlation test or the Kruskal-Wallis rank-sum test.

A *P*-value <.05 was considered statistically significant. We organized and managed the data using Microsoft Excel (Microsoft Corp, Redmond, WA) and analyzed it using SPSS 29.0.1 (IBM Corporation, Armonk, NY).

### Ethics Review

Approval for this international MSU study was obtained from the institutional review board at Tsukuba Medical Center Hospital.

## RESULTS

### Active Mobile Stroke Unit Programs

#### Overview

Of the 33 active MSU programs contacted, we received 19 (58%) e-mail replies. Of these, 12 programs were in North America, four in Asia/Oceania, and three in Europe (Table 1). The operation of four programs was on hold due to the COVID-19 pandemic (three in North America, one of which was delayed due to financial difficulties and one in Europe due to a planned program shift. Two programs, one in North America and another in Asia, had a whole-body CT scanner onboard in their MSUs. One program in North America did not use a telemedicine system. One program in North America and another in Europe had three MSUs, while one in Asia had six MSUs. Post-survey investigation found that of the 19 responding programs, 12 reported the number of patients treated per a single MSU per year, ranging from 52 to 1,663 (median 781). (Data of seven programs could not be found in the original articles or on their official websites.)

#### Status of Active Mobile Stroke Unit Programs

**Figure 1:**
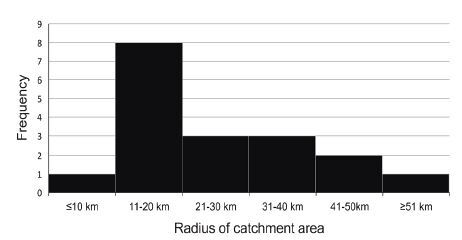
Histogram of catchment area of mobile stroke units (MSU). Data shown are for a single MSU in a program. Data were obtained from 18 programs. *km*, kilometer.

The models of onboard CT scanners were as follows: CereTom (NeuroLogica [https://www.neurologica.com/]) in 14 programs (74%); OmniTom (NeuroLogica) in two (11%); CereTom and OmniTom in one (5%); SOMATOM Scope (SIEMENS Healthineers [https://www.siemens-healthineers.com/]) in one (5%); and Ingenuity (Philips [https://www.philips.com/] in one (5%). The hours of operation varied among programs (Table 2). Nine (47%) operated five weekdays per week, while the remaining 10 (53%) operated seven days per week. The most common time of operation was eight business hours every weekday in five programs (26%). Four programs (21%) reported operating 24 hours/day, seven days/week. The number of MSU staff on board ranged from two to seven, with the mode of four reported by six programs (32%). Six programs (32%) had no physicians on board. The most commonly reported number of physicians was one (58%); the physicians were typically vascular neurologists or stroke physicians, as reported by eight programs (48%). An expert nurse was on board in 12 (63%), a radiology technician in 16 (84%), one or two emergency medical technicians in nine (48%), one or two paramedics in 11 (58%), and one telemedicine technologist in two (11%). The catchment area radius for MSU operation ranged from 5–250 kilometers (km), with a median value of 22 km and a skewness of 3.9 (n = 18; Figure 1).

**Figure 2:**
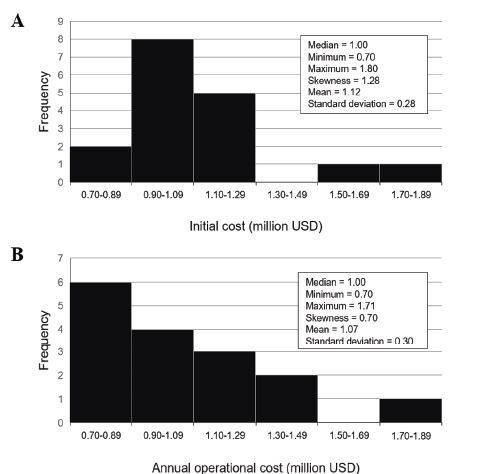
Costs of mobile stroke units (MSU). Data on initial costs (A) and annual operating costs (B) were obtained from 17 and 16 programs, respectively. The cost is estimated for a single MSU. USD, United States dollar.

The costs associated with the MSU programs are presented in histograms in Figure 2. The initial costs of 17 programs ranged from 0.7–1.8 million United States dollars (USD), with a median value of 1.0 million USD and a skewness of 1.3 (Figure 2A). The annual operation costs of 16 MSU programs ranged from 0.7 to 1.7 million USD, with a median value of 1.0 million USD and a skewness of 0.7 (Figure 2B). The detailed financial statuses of 17 MSUs are shown in Table 3. The financial sources for the MSU programs consisted of government funds in eight programs (47%), non-governmental foundation funds in four (24%), hospital organization funds in eight (47%), philanthropic funds in 10 (59%), and reimbursement in eight (47%). Only two programs (12%) received full reimbursement. The most commonly used financial strategy was a philanthropic fund only in three programs (17%). The combination of all five financial sources in two MSU programs (12%) was the next most common strategy, while the remaining programs all used a different type (1 program, 6%, each). The gross financial balance for 15 programs was positive in four programs (27%), negative in eight (53%), and neutral in three (20%).

The reported challenges faced by 18 programs (Table 4) were finances in 17 programs (94%), human resources in 10 (56%), maintenance in three (17%), and follow-ups on patient outcomes in one (6%). The most frequent free-text comments from 15 programs (Table 4) were related to insufficient or absent reimbursement in nine programs (60%). Other comments included MSU replacement, vehicle wear-and-tear, operational troubles due to harsh weather, service suspension due to maintenance, and buy-in or expansion of the catchment area (two programs, 13%, each). No significant differences or correlations were found among the operational and financial variables relevant to MSUs.

Table 5 presents the the difference in demographic, economic, and healthcare backgrounds between active MSU and non-MSU regions. Compared to non-MSU regions, active MSU regions (MSU group) had higher population size at the country, state, and city levels. Similarly, Mthe SU group had higher population density at the state level, and nominal GDP was higher at both the country and state levels. Furthermore, the active MSU group reported higher HAQ index at the country level and physician density at both the country and state levels. Additionally, the active MSU group reported a lower age-standardized stroke incidence rate and age-standardized stroke DALY rate at the country level.

**Figure 3:**
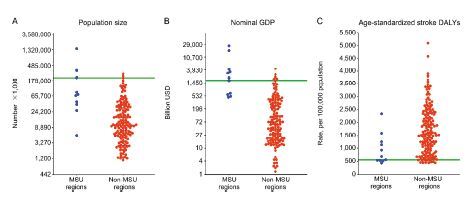
Scattergram showing the data of (A) population size, (B) nominal gross domestic product, and (C) age-standardized stroke disability-adjusted life year rate at the country level. *MSU*, mobile stroke unit; *GDP*, gross domestic product; *DALY*, disability-adjusted life year; *USD*, United States dollar.

We identified the threshold between active MSU and non-MSU regions at the country level as follows: threshold (predictive probability of overall model quality, predictive accuracy rate); population size (216,927×1,000 [0.72, 0.94]), nominal GDP (670 billion USD [0.91, 0.94]), and the age-standardized stroke DALY rate (562 per 100,000 [0.62, 0.86]) (Figure 3).

#### Relationship Between Practical Status and Social Background of Mobile Stroke Units

**Figure 4:**
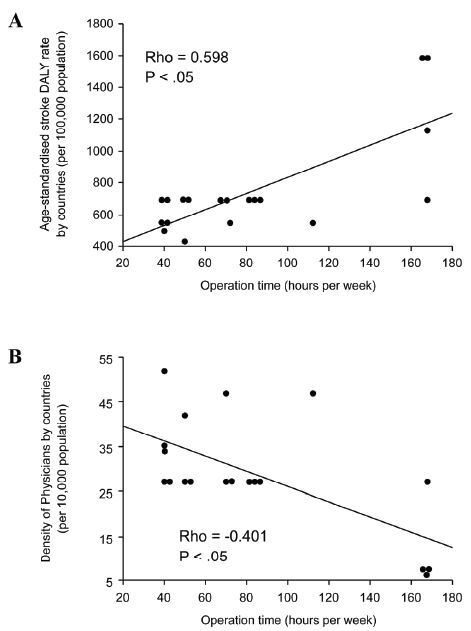
Scattergram showing the correlation between the mobile stroke unit hours of operation per week (MSU) and the age-standardized rate, disability-adjusted life year, of all strokes by country (A) and between the MSU hours of operation per week and physician density by country (B) disability-adjusted life year. *DALY*, disability-adjusted life year.

In active MSU programs, a significant positive correlation was found between hours of operation per week and age-standardized stroke DALY rate by country (ρ = 0.598, *P* < .05, Figure 4A). In addition, a significant negative correlation was found between hours of operation per week and physician density by country (ρ = −0.401, *P* < .05, Figure 4B). Otherwise, no significant differences or correlations were found in active MSU programs between variables of the practical status and social background.

## DISCUSSION

The 58% survey response rate (19 programs) was comparable to that of previous surveys, such as the 2018 review paper by Calderon 28 that summarized the operational status of MSUs in 14 global regions, the 2021 survey report by Reichenbach 29 that showed reimbursement limitations and a negative financial status of 19 programs in the United States, and the 2023 global survey report by Kovi 30 that clarified the impact of the COVID-19 pandemic on MSU operations in 20 programs. The number of patients treated with MSUs in our survey exhibited a considerable degree of variability. This result seems to be contingent on the population of the MSU coverage area, although this data is not presented.

Regarding the operational status of MSUs, this survey found that the CereTom (NeuroLogica) was the most common onboard CT scanner. It is the smallest and lightest among all scanners used at active MSU sites, and is expected to save costs 31 and to work well in new regions, particularly rural or low economic areas. The hours of operation varied from program to program, with most MSUs limiting their operations to daytime on weekdays. Just one-fifth of all sites operated 24 hours/day, 7 days/week. These findings are consistent with those of previous reports and likely reflect the importance of maintaining a cost–performance balance. 28 The onboard staff consisted of a combination of various professionals. Notably, no physician was on board in one-third of the programs. This is probably due to the advanced qualifications held by the non-physician staff and the use of telemedicine, as has been previously reported. 31 The catchment areas reported, which ranged from 5–250 km, were consistent with previous reports 31 and indicate that MSU coverage areas are program- or region-specific, ranging from the city to the state level.

Regarding the financial status of MSUs, the initial costs and annual operating costs reported in this study were high, consistent with previous studies. 4,10,31 The annual operating costs consisted primarily of personnel, MSU vehicle or CT scanner maintenance, and telemedicine use. 10 To reduce costs, MSU staff need to have more advanced qualifications. Moreover, the development of cheaper or more cost-effective MSU hardware is necessary. 31 Most MSU programs relied on philanthropic funds for financial support, with some programs being solely supported by philanthropic funds. Full reimbursement was received in very few programs, meaning that most programs had a negative gross financial balance. Accordingly, financial issues were commonly raised as a challenge confronted by MSU programs. These results are in line with a 2021 report, which showed that the majority of MSU sites in the United States are at financial risk, 29 and with a 2022 report that proposed specific solutions for this issue. 10 The second most frequently encountered challenge was related to human resources. Human resources is a critical issue when operating MSUs, especially in remote or rural areas, 13 and when implementing a telemedicine system. 32

When comparing regions with and without active MSUs in terms of their demographic and economic contexts, regions with MSUs had a larger population at the country, state, and city levels and had larger nominal country and state GDP. A large population influences the economic development of a society. 33 Nominal GDP is the total market value of all goods and services produced in a country’s economy in a given period and is just one way to measure the economic performance of a country. 34 Therefore, our results suggest that most MSUs were implemented under the advanced economic conditions at the three administrative levels. Additionally, regions with active MSUs had a higher population density at the state level. This makes sense in light of our finding that the MSU catchment area extends to the state level (Figure 1) and may be explained by the idea that high population density can facilitate the development of infrastructure. 35

In our investigation of the healthcare background of regions with and without MSUs, we found that at the country level, regions with MSUs exhibited a lower stroke-incidence rate, and a lower stroke DALY rate, as well as a higher HAQ index. The disease incidence is a commonly used measure of the disease risk for a specific population during a specified period. 36 The DALY is an indicator of the disease burden and represents the sum of years of life lost and years lived with disability. 37 The HAQ index estimates healthcare access and quality across different locations. This index is based on death rates and mortality-to-incidence ratios of various causes of death, including stroke (cerebrovascular disease). 26 Therefore, our results suggest that current MSUs operate in areas with an advanced healthcare system and a lower disease burden at the country level. Regions with MSUs also exhibited a higher physician density both at the country and state levels. Physician density, which represents the number of physicians per population, is a crucial measure of the health human resources necessary for the healthcare system to function properly. 38 An adequate supply of physicians is necessary to ensure access to affordable and quality healthcare. Our results suggest that the success of MSU operations may rely on the presence of better health human resources in the vicinity of the MSU catchment area.

The country-level thresholds for distinguishing between regions with and without MSUs were dependent on the population, nominal GDP, and age-standardized stroke DALY rate. These thresholds may be useful, approximate indicators to understand the current status of MSU implementation. Since population and nominal GDP data are updated annually and always available, these two thresholds may be particularly practical. With regard to the stroke DALY rates, the range between the threshold and the minimum value appears narrow (Figure 3C). Thus, this threshold may not be a practical approximate indicator.

Furthermore, we found a significant positive correlation between the MSU hours of operation and the country’s age-standardized stroke DALY rate. This supports the notion that the DALY metric reflects the degree of unmet medical needs. 39 We also found a significant negative correlation between the MSU hours of operation and the country’s physician density. Reports have suggested that a shortage of physicians presents a significant barrier to providing effective and equitable healthcare services. 40 In such regions, physicians may need to be sourced from other areas to participate in the MSU program. Otherwise, it may be necessary to use telemedicine systems to their fullest potential. Taken together, the hours of operation of MSUs may depend on insufficient healthcare delivery systems or environments where stroke is more likely to occur.

## LIMITATIONS

This study has several limitations and problems. First, the survey response rate (58%) was relatively low in the field of medicine and healthcare. The majority of non-responding MSU programs (10) were located in the United States (71% of all non-response programs, Table 1). It can be confirmed via official websites that in the United States non-responding programs and responding programs employ nearly identical models of CT scanners or MSU vehicles. Consequently, non-response bias in our survey is minimal. Second, we did not inquire about the details of MSU costs or finances. Questions were simplified to maximize the number of responses. Our results showed that the total costs and model of CT scanner used were almost the same as those reported by previous studies. This means that the remaining costs including personnel and maintenance have probably not changed significantly from before, but there is no more specific information available regarding costs such as vehicle repair, CT maintenance, or telemedicine usage among MSU programs.

We also found that only a few MSU sites were fully reimbursed or had a positive gross financial balance. However, we were unable to obtain the essential details necessary to offer solutions to the financial issues faced by the majority of MSU sites. Considering these limitations, future studies should focus on the specifics of MSU costs and finances. Third, data on nominal GDP, stroke incident rate, stroke disability-adjusteds life year rate, HAQ index, and physician density were unavailable at the city level or at both state and city levels for all locations. Therefore, we could not form conclusions on the regional specificity of economic and healthcare backgrounds in the states and cities. These factors should be analyzed in individual studies. Fourth, the limited number of active MSU programs prevented us from conducting a proper multivariate analysis or a two- or three-way Mann-Whitney U analysis. Thus, our study did not demonstrate any interactions on the relationships between MSU status variables, or interactions on the relationships between regions with and without MSUs at the country (three-way) and state (two-way) levels.

Finally, we did not inquire about the impact of the COVID-19 pandemic on MSU operations. Kovi 30 reported that MSUs were able to overcome the challenges posed by the early phase of the COVID-19 pandemic. A retrospective study would clarify the impact of the entire phase of COVID-19 pandemic on MSUs. This information is essential for MSUs to prepare for any future pandemics.

## CONCLUSION

Mobile stroke units operate globally in program- or region-specific ways. The MSU programs are confronted with significant financial challenges, including high start-up and annual operating cost or uncertain reimbursement. Regions with MSUs tend to have relatively large populations, nominal GDP, HAQ index, and physician density, as well as relatively low stroke-incidence rates and stroke disability-adjusted life year rates. Meanwhile, regions without MSUs appear to have significant barriers to MSU implementation with regard to their demographic, economic, and healthcare backgrounds. Nevertheless, for the populations in these regions, MSU-based care appears to be a more beneficial option, particularly considering the disease burden. To successfully implement an MSU program in a new region, it is essential that the project team conduct a thorough assessment of the regional social contexts and address any pertinent issues. Finally, strategies for establishing reimbursements and reducing costs should be considered.

## Figures and Tables

**Table 1:** Overview of mobile stroke unit programs.

Location	All active MSU programs, 8 n	Programs that responded to the survey		

		Number, n (% of active programs)	Operating as of March 2023, n (% of respondents)	MSU vehicles per program, median (range)
North America	22	12 (55)^*^, ^**^, †, ‡	9 (75)	1 (1–3)
Asia/Oceania	5	4 (80)†	4 (100)	1 (1–6)
Europe	4	3 (75)^***^	2 (67)	1 (1–3)
South America/Africa	2	0 (0)	N/A	N/A
Total	33	19 (58)	15 (79)	1 (1–6)

Four programs were put on hold in March 2023, In these cases, the data provided were from the period when the MSU had most recently operated.

† Two programs had a whole-body CT scanner onboard.

‡ One program did not employ a telemedicine system for MSU.

*MSU*, mobile stroke unit; *CT*, computed tomography; *N/A*, not assessed.

**Table 2:** Operation characteristics of mobile stroke units.

Demographics	Frequency	% of total
Operation time (n=19)		
5 days per week (weekdays)	9	47
8 hours, daytime	5	26
10 hours, daytime	4	21
7 days per week	10	53
10 hours, daytime	2	11
12 hours, daytime	3	16
16 hours, daytime to late evening	1	5
24 hours	4	21

Onboard personnel (n=19)		
Total number		
2	5	26
3	3	16
4	6	32
5	4	21
7	1	5
Physician		
0	6	32
1	11	58
Vascular neurologist/stroke physician	8*	48
Neuroradiologist	2	5
Trained medical doctor	1	5
2		
Vascular neurologist, radiologist	1	5
3		
Emergency physician, radiologist, anesthesiologist	1	5
Expert nurse		
0	7	37
1	12**	63
Radiology technician		
0	3	16
1	16†	84
Emergency medical technician		
0	10	52
1	6	32
2	3	16
Paramedic		
0	8	42
1	8	42
2	3	16
Telemedicine technologist		
0	17	89
1	2	11

* One vascular neurologist had the qualifications of an emergency physician.

** One held a doctorate and underwent stroke fellowship training.

† One had received training as an emergency medical technician.

**Table 3:** Financial status of mobile stroke units.

Category	Subcategory	Financial source/balance	Frequency	% of total
Finance source (n=17)	Included	GF	8	47
		NGFF	4	24
		HOF	8	47
		PF	10	59
		RI	8	47
		Partial	6	35
		Full	2	12
	Individual	GF only	1	6
		GF, HOF	1	6
		GF, RI	1	6
		GF, NGFF, HOF, PF, RI	2	12
		NGFF, HOF	1	6
		NGFF, RI	1	6
		HOF only	1	6
		HOF, RI	1	6
		PF only	3	17
		PF, GF	1	6
		PF, HOF	1	6
		PF, GF, RI	1	6
		PF, NGFF, RI	1	6
		PF, HOF, RI	1	6
Gross financial balance (n=15)		Positive	4	27
		Negative	8	53
		Neutral	3	20

*GF*, government funds; *NGFF*, non-governmental foundation funds; *HOF*, hospital organization funds; *PF*, philanthropic funds; *RI*, reimbursement.

**Table 4:** Present challenges confronted and free comments regarding mobile stroke units.

Category	Frequency (%, n = 18)	Free Comment	Frequency (%, n = 15)
Finance	17 (94)	Insufficient or absent reimbursement*	9 (60)
		Cost of MSU replacement	2 (13)
		Others	6 (40)
Human resources	10 (56)		
Maintenance	3 (17)	Vehicle wear-and-tear	2 (13)
		Out of service during maintenance	1 (7)
Patients’ outcomes	1 (6)	Follow-up of patients’ outcome	1 (7)
Others	6 (33)	Out of service due to harsh weather	2 (13)
		Buy-in or expansion of catchment area	2 (13)
		Extension of operation hours	1 (7)
		Stroke education for community residents	1 (7)

* Data included fragmentation of reimbursement due to the COVID-19 pandemic (n=1).

**Table 5:** Demographic, economic, and healthcare backgrounds of regions with and without active mobile stroke units.

Categories	Measures	Results		

		With MSU	Without MSU	*P*-value
Population size, number (×1,000)				
Countries 16	N	11	150	<.001†
	Mean (SD)	339,822 (543,541)	28,131 (43,858)	
	Median (range)	71,697 (5,434–1,425,887)	11,062 (1,120–275,501)	
States^17^,^18^,^19^	N	24	289	<.01‡
	Mean (SD)	17,116 (21,938)	7,048 (16,227)	
	Median (range)	6,348 (1,629–83,720)	3,189 (423–39,030)	
Cities^18^,^19^,^20^	N	33	480*	<.05‡
	Mean (SD)	1,337 (1,900)	193 (436)	
	Median (range)	677 (16–5,588)	93 (10–540)	
Population density, per km^2^				
Countries 21	N	11	150	.94†
	Mean (SD)	137 (138)	242 (886)	
	Median (range)	136 (3–416)	80 (2–8,286)	
States 17,18,19	N	24	276	<.05‡
	Mean (SD)	1,129 (3,211)	330 (1,001)	
	Median (range)	175 (7–3,623)	122 (3–310)	
Cities^18^,^19^,^22^	N	33	480*	0.17‡
	Mean (SD)	2,961 (3,459)	1,560 (1,924)	
	Median (range)	1,795 (270–15,378)	938 (143–6,338)	
Nominal GDP, billion USD				
Countries 23	N	11	145	<0.001†
	Mean (SD)	5,450 (8,308)	287 (578)	
	Median (range)	2,140 (477–25,463)	70 (2–4,231)	
	N	24	291	
States^18^,^24^	Mean (SD)	706 (850)	142 (268)	<.01‡
	Median (range)	174 (19–844)	63 (3–432)	
Age-standardized incidence rate of all strokes, per 100,000 population				
Countries 9	N	11	149	<.05†
	Mean (SD)	116 (39)	137 (34)	
	Median (range)	106 (83–226)	129 (79–224)	
Age-standardized DALYs rate of all strokes, per 100,000 population				
Countries 25	N	11	149	<.01†
	Mean (SD)	953 (594)	1706 (950)	
	Median (range)	692 (428–2,342)	1543 (338–5,091)	
HAQ index				
Countries 26	N	11	148	<.01†
	Mean (SD)	71 (21)	54 (20)	
	Median (range)	81 (32–91)	54 (15–93)	
Density of physicians, per 10,000 population				
Countries 27	N	11	147	<.05†
	Mean (SD)	29 (17)	18 (16)	
	Median (range)	35 (3–52)	13 (1–84)	
States^18^	N	24	282	<.05‡
	Mean (SD)	4.3 (4.7)	2.2 (4.7)	
	Median (range)	1.5 (0.1–20.5)	0.8 (0.1–9.9)	

Countries were defined as having a population of at least one million. States included regions (administrative districts), provinces, and counties. Cities included the smallest administrative districts in cases where city data was unavailable. Non-MSU region data at the state or city level was selected from the same country or state as the MSU.

* The top 20 most populous cities were analyzed. The median is the median of the medians at the state level and the median of the medians of the medians at the city level. Only key references were provided due to the limitation of the number of references.

† Mann-Whitney U test,

‡ Wilcoxon signed rank test using mean-ranks at the state level and mean-ranks of mean-ranks at the city level.

*MSU*, mobile stroke unit; *SD*, standard deviation; *USD*, United States dollar; *GDP*, gross domestic product; *DALY*, disability-adjusted life year; *N/A*, not assessed.

## References

[1] Ebinger M, Siegerink B, Kunz A (2021). Association between dispatch of mobile stroke units and functional outcomes among patients with acute ischemic stroke in Berlin. JAMA.

[2] Grotta JC, Yamal J-M, Parker SA (2021). Prospective, multicenter, controlled trial of mobile stroke units. N Engl J Med.

[3] Kim J, Easton D, Zhao H (2021). Economic evaluation of the Melbourne Mobile Stroke Unit. Int J Stroke.

[4] Oliveira Gonçalves AS, Rohmann JL, Piccininni M (2023). Economic evaluation of a mobile stroke unit service in Germany. Ann Neurol.

[5] Zhao H, Coote S, Easton D (2020). Melbourne mobile stroke unit and reperfusion therapy: greater clinical impact of thrombectomy than thrombolysis. Stroke.

[6] Cooley SR, Zhao H, Campbell BCV (2021). Mobile stroke units facilitate prehospital management of intracerebral hemorrhage. Stroke.

[7] Walter S, Kostpopoulos P, Haass A (2010). Bringing the hospital to the patient: first treatment of stroke patients at the emergency site. PLoS One.

[8] Fassbender K, Lesmeister M, Merzou F (2023). Prehospital stroke management and mobile stroke units. Curr Opin Neurol.

[9] GBD 2019 Stroke Collaborators (2021). Global, regional, and national burden of stroke and its risk factors, 1990–2019: a systematic analysis for the Global Burden of Disease Study 2019. Lancet Neurol.

[10] Navi BB, Audebert HJ, Alexandrov AW (2022). Mobile stroke units: evidence, gaps, and next steps. Stroke.

[11] Hiroki M, Kohno Y, Misawa M (2023). Update: mobile stroke unit and its on-board diagnostic imaging. Jpn J Stroke.

[12] Walter S, Fassbender K, Easton D (2021). Stroke care equity in rural and remote areas - novel strategies. Vessel Plus.

[13] Olatunji G, Kokori E, Isarinade T (2023). Revolutionizing stroke care in Africa: a mini review of the transformative potential of mobile stroke units. Medicine (Baltimore).

[14] Bagot KL, Purvis T, Hancock S (2023). Interdisciplinary interactions, social systems and technical infrastructure required for successful implementation of mobile stroke units: a qualitative process evaluation. J Eval Clin Pract.

[15] Hiroki M, Kohno M, Misawa M (2022). Current problems and future prospects for Japan’s mobile stroke units. J Jpn Soc Emer Med.

[16] United Nations (2023). World population prospects.

[17] National Institute of Statistics and Censuses (INDEC) (2022). 2022 Census: national census of population, households and housing.

[18] Statista (2025). The Statistics portal for market data, market research and market studies.

[19] City Population (2025). Population statistics in maps and charts for cities, agglomerations and administrative divisions of all countries of the world.

[20] Census & Demographic Data Provider – Cubit (2025). Find your best zip codes, cities or counties with the most current, easy-to-use demographic data delivered instantly.

[21] Pison G (2019). The population of the world (2019). Population & Societies.

[22] USA.com (2025). Rankings Search.

[23] International Monetary Fund (2025). GDP, current prices.

[24] OECD Statistics (2025). Regions and cities: regional statistics; regional economy; gross domestic product, small regions TL3 (territorial level 3).

[25] Krishnamurthi RV, Ikeda T, Feigin VL (2020). Global, regional and country-specific burden of ischaemic stroke, intracerebral haemorrhage and subarachnoid haemorrhage: a systematic analysis of the global burden of disease study 2017. Neuroepidemiology.

[26] GBD 2019 Healthcare Access and Quality Collaborators (2022). Assessing performance of the Healthcare Access and Quality Index, overall and by select age groups, for 204 countries and territories, 1990–2019: a systematic analysis from the Global Burden of Disease Study. Lancet Glob Health.

[27] GBD 2019 Universal Health Coverage Collaborators (2020). Measuring universal health coverage based on an index of effective coverage of health services in 204 countries and territories, 1990–2019: a systematic analysis for the Global Burden of Disease Study 2019. Lancet.

[28] Calderon VJ, Kasturiarachi BM, Lin E (2018). Review of the mobile stroke unit experience worldwide. Interv Neurol.

[29] Reichenbach K, Mathiesen C, Thomas L (2021). Reimbursement of mobile stroke units in the United States: a survey by the Prehospital Stroke Treatment Organization (PRESTO). Stroke.

[30] Kovi S, Blaginykh E, Buletko AB (2023). The early impact of COVID-19 pandemic on mobile stroke unit care delivery: a worldwide survey. Clin Neurol Neurosurg.

[31] Fassbender K, Merzou F, Lesmeister M (2021). Impact of mobile stroke units. J Neurol Neurosurg Psychiatry.

[32] Demaerschalk BM, Berg J, Chong BW (2017). American Telemedicine Association: telestroke guidelines. Telemed J E Health.

[33] Atanda AA, Aminu SB, Alimi OY (2012). The role of population on economic growth and development: evidence from developing countries. MPRA Paper.

[34] The Investopedia Team (2024). Nominal gross domestic product: definition and how to calculate.

[35] Yegorov Y (2015). European Regional Science Association (ERSA). Economic role of population density.

[36] Tenny S, Boktor SW (2023). Incidence.

[37] World Health Organization (2020). Disability-adjusted life years (DALYs).

[38] GBD 2019 Human Resources for Health Collaborators (2022). Measuring the availability of human resources for health and its relationship to universal health coverage for 204 countries and territories from 1990 to 2019: a systematic analysis for the Global Burden of Disease Study 2019. Lancet.

[39] Harrison JD, Young JM, Butow PN (2013). Needs in health care: what beast is that?. Int J Health Serv.

[40] WHO: Global Health Workforce Alliance (2013). A universal truth: no health without a workforce.

